# Serum Amino Acid and Fatty Acid Metabolites as Predictors of Sleep Disorders in Children: A Risk Prediction Model

**DOI:** 10.3390/biomedicines14030546

**Published:** 2026-02-27

**Authors:** Liuyan Zhu, Dan Yao, Lei Wang, Tianmiao Gu, Weijun Chen

**Affiliations:** Department of Pediatric Health Care, Children’s Hospital, Zhejiang University School of Medicine, National Clinical Research Center for Children and Adolescents’ Health and Diseases, Hangzhou 310052, China; zhuliuyan@zju.edu.cn (L.Z.);

**Keywords:** sleep disorders, children, amino acids, fatty acids, risk prediction model

## Abstract

**Objective**: Adequate sleep is vital for children’s growth and well-being. This study investigates serum amino acid and fatty acid metabolic indicators in children with sleep disorders, identifies independent factors, and develops a predictive model. **Methods**: A total of 143 children diagnosed with sleep disorders (*n* = 143) were compared to 120 typically developing children (*n* = 120). Serum levels of 12 amino acids and 7 fatty acids were measured using liquid chromatography–tandem mass spectrometry. Differences between groups were assessed using *t*-tests or Mann–Whitney U tests. Independent factors were identified via multivariate logistic regression, leading to the construction of a predictive model. Its diagnostic efficacy was evaluated through receiver operating characteristic analysis, calibration curves, and decision curve analysis (DCA). Subgroup analysis of different sleep disorder subtypes was also performed to explore metabolic characteristic differences. **Results**: Significant differences in multiple metabolic indicators were found (*p* < 0.05) between these two groups. Seven amino acids were elevated, including glutamine and tryptophan, while linoleic acid and taurine levels were reduced. Analysis of four sleep disorder subtypes revealed no significant differences in most metabolic indicators among subtypes, with only taurine levels showing notable heterogeneity, the highest in parasomnia and the lowest in insomnia. Multivariate analysis revealed that arachidonic acid (OR = 0.75, 95% CI: 0.649–0.866), the ratio of cerotic acid to behenic acid (OR = 0.39, 95% CI: 0.186–0.816), aspartic acid (OR = 1.1, 95% CI: 1.040–1.164), glutamine (OR = 1.009, 95% CI: 1.004–1.014), taurine (OR = 0.985, 95% CI: 0.974–0.995), and phenylalanine (OR = 1.047, 95% CI: 1.018–1.078) were identified as independent factors for the development of sleep disorders (*p* < 0.05). The predictive model achieved the area under the ROC curve of 0.935 (95% CI: 0.904–0.967), with a threshold of 0.748 yielding sensitivity of 0.881 and specificity of 0.867. Ten-fold cross-validation confirmed robust generalizability (AUC: 0.927–0.916), and adjustable thresholds enabled flexible clinical application. Calibration curves and DCA demonstrated good agreement and clinical utility. **Conclusions**: Children with sleep disorders exhibit notable serum metabolic disturbances. The developed predictive model provides high diagnostic value and practicality for early screening and targeted interventions.

## 1. Introduction

High-quality sleep is fundamental for the growth and development of children, as well as for their overall psychological and physical health. It is crucial for enhancing cognitive functions, regulating emotional states, and sustaining a robust immune system. Recent data suggests that around 25% of children experience sleep disorders [1], with those having comorbid chronic conditions or neurodevelopmental disorders at an even greater risk. Further research by Tamir S indicates that the prevalence of sleep disorders among normally developing children can be as high as 20% [2]. Poor sleep quantity or quality may lead to various negative consequences, including delayed growth, unpredictable emotional changes, and a significantly elevated risk of obesity. Among adolescents, these concerns may also surface as issues related to attention deficits, anxiety, and depression [3,4,5,6,7]. Thus, the early detection and timely management of sleep-related difficulties in children are critical for promoting healthy development. Existing clinical approaches to tackle pediatric sleep issues encompass sleep hygiene education, behavioral therapies, mindfulness interventions, and melatonin pharmacotherapy [8,9]. However, the effectiveness of these interventions tends to vary considerably due to individual patient differences. On the diagnostic side, current methodologies have notable limitations. Frequently used assessment instruments, such as the Children’s Sleep Habits Questionnaire (CSHQ) [10], rely heavily on subjective reports that can be affected by parental perception and observational biases. Polysomnography, viewed as the “gold standard” for sleep disorder diagnosis, requires comprehensive overnight monitoring in specialized sleep laboratories, posing complexities in execution and high costs for equipment [11,12]. This situation restricts its broader application in primary care and large-scale screening programs. In light of these challenges, there is an urgent need to develop an objective, user-friendly, and cost-effective predictive tool within the field of clinical research focused on pediatric sleep disorders.

Children’s sleep exhibits a rhythmic pattern of alternating deep and light sleep, regulated by a complex mechanism controlling sleep–wake transitions. This mechanism comprises two core components: firstly, the interplay between wake-promoting and sleep-promoting neurons forms a “switch” system that directly mediates sleep–wake behaviors. Secondly, the transitions between wakefulness and different sleep stages depend on the precise modulation of neurochemical substances in the brain. Notable wake-promoting molecules include orexin, serotonin, norepinephrine, histamine, dopamine, and epinephrine, while gamma-aminobutyric acid (GABA), adenosine, and prostaglandins support non-rapid eye movement (NREM) sleep, and glutamate is essential for regulating rapid eye movement (REM) sleep [13,14,15]. Recent advancements in metabolomics have shed light on the connection between metabolic indicators and sleep regulation. Amino acids, which are crucial precursors for neurotransmitter synthesis, can affect the excitability balance of the central nervous system if their metabolism is disrupted [16]. Fatty acids, as fundamental constituents of neuronal cell membranes, contribute to neurotransmission and help regulate inflammatory responses [17]. Previous research has demonstrated that a deficiency in docosahexaenoic acid (DHA) is significantly linked to immature sleep patterns in neonates [18]. However, existing studies tend to concentrate on the relationship between single metabolic indicators and sleep disorders, lacking a systematic exploration of combined predictive models that incorporate multiple indicators. Moreover, the clinical utility of these models has not been thoroughly assessed.

Considering the current state of research and clinical requirements, this study is intended to systematically examine serum amino acid and fatty acid metabolic profiles to pinpoint independent predictive factors related to sleep disorders in children. We aim to construct and thoroughly validate a risk prediction model that offers an efficient and objective early screening tool for clinical practice. Moreover, this investigation seeks to lay a solid theoretical groundwork for precise interventions aimed at addressing pediatric sleep disorders.

## 2. Materials and Methods

### 2.1. Study Subjects

This is a case–control study. The case group included children who were naturally admitted to the Sleep Disorder Specialty Clinic of the Department of Pediatric Health Care in our hospital from June 2024 to June 2025. These children presented with sleep-related symptoms such as difficulty falling asleep, bruxism, and night screaming. All cases with sleep-related symptoms were initially enrolled for further assessment via standardized parental interviews and symptom inquiry, and the final diagnosis was made in accordance with the criteria defined in the International Classification of Sleep Disorders, Third Edition (ICSD-3) [1].

Inclusion Criteria: Children aged 3 to 8 years; Diagnosed with one of the following sleep disorder subtypes based on the ICSD-3: sleep-disordered breathing (including obstructive sleep apnea), parasomnia (including sleepwalking and night terrors), insomnia, and sleep-related movement disorders; Duration of symptoms ≥ 1 month (verified through structured parental interviews using a standardized protocol); Informed consent obtained from the guardians of the child, who also signed an informed consent form.

Exclusion Criteria: Presence of severe cardiovascular, hepatic, renal, or other organ diseases, including neurological disorders (e.g., autism spectrum disorder, attention deficit hyperactivity disorder); Existence of metabolic disorders (e.g., diabetes, congenital metabolic defects), epilepsy, cerebral palsy, intellectual disabilities, genetic abnormalities, or psychiatric illnesses; Administration of pharmacological treatments for sleep disorders within the past month; Regular use of supplements that may affect serum metabolites (e.g., omega-3 fatty acids, multivitamins, GABA supplements) within the past month.

Additionally, 120 children undergoing routine health check-ups at the routine pediatric health examination clinic of our hospital during the same period were selected as the control group.

Control Group Inclusion Criteria: No reported sleep-related symptoms, as determined by parental interviews; Normal neurodevelopment and behavioral status, confirmed by routine pediatric health check-up tools: Ages and Stages Questionnaires (ASQ), Peabody Picture Vocabulary Test (PPVT), and Conners’ Parent Rating Scale-Revised (CPRS-R), with all scores within the normal reference range; No history of psychiatric illnesses, neurological disorders, or other physical illnesses; Informed consent obtained from the guardians.

This study included 6 independent predictive factors, and the ratio of events (number of children with sleep disorders, *n* = 143) to variables was approximately 24:1 (143/6), which reflects that the sample size meets the recommended standards for such analyses and ensures the stability of the model.

This study received approval from the hospital’s ethical committee (Ethics Approval Number: 2024-IRB-0391-P-01). No additional compensation was provided to the participants, as all detection items were essential assessment contents required for routine pediatric physical examinations or clinical diagnosis and treatment. Guardians voluntarily agreed to the blood tests based on the need for monitoring their children’s health status, and children whose guardians refused blood collection were excluded from the study in accordance with the exclusion criteria.

### 2.2. Materials & Methods

#### 2.2.1. General Data Collection

General data, including gender, age, body mass index (BMI) of the two groups of children, were collected through face-to-face interviews combined with a review of electronic medical records.

#### 2.2.2. Detection of Serum Metabolic Indicators

The 12 amino acids and 7 fatty acids detected in this study were preselected based on their established roles in sleep regulation (neurotransmitter synthesis, neuronal membrane function, and inflammatory modulation) and prior literature evidence. All are part of the routine “neurotrophic factor mass spectrometry detection” in our department, ensuring reliable detection performance. Considering the physiological characteristics of children, fasting for 4–6 h before blood collection was defined as the fasting standard, and the last meal time of each child was confirmed through standardized inquiry to ensure consistency in metabolic markers and minimize variability. All blood samples were collected within a fixed time window (8:00–10:00 AM) to minimize the impact of circadian rhythms on metabolite levels. Samples were collected in the same phlebotomy room of the Department of Pediatric Health Care by uniformly trained professional phlebotomists, following standard procedures and using gold-top serum separator tubes to ensure standardized collection processes. To ensure sample quality, all specimens were processed within two hours of collection. The detection of amino acids, including aspartic acid, glutamine, glutamic acid, glycine, serine, kynurenine, tyrosine, GABA, taurine, tryptophan, and phenylalanine, alongside fatty acids, such as linoleic acid (LA), eicosapentaenoic acid (EPA), DHA, arachidonic acid (ARA), cerotic acid, behenic acid, and the ratio of cerotic acid to behenic acid concentrations, was performed using the API 4500 liquid chromatography–tandem mass spectrometry (LC-MS/MS) system (Triple Quad™ 4500MD, AB Sciex, Framingham, MA, USA). Detailed procedures for sample pretreatment (including amino acid and fatty acid extraction), chromatographic separation conditions (column type, mobile phase composition, flow rate, column temperature), and mass spectrometry parameters (ionization mode, ion source temperature, electrospray voltage, multiple reaction monitoring (MRM) ion pairs) are provided in the Supplementary Materials.

Optimal mass spectrometry acquisition conditions and corresponding MRM ion pairs for each fatty acid were determined using single standard solutions, allowing for qualitative identification based on the retention times and characteristic ion pairs of the analytes and their internal standards. To ensure reproducibility and inter-batch comparability, each batch of analyses included low, medium, and high concentrations of quality control samples. The measured values of quality control samples all fell within ±2 standard deviations of the target value, with between-batch coefficients of variation (CV) ≤ 10%. All samples contained internal standards to correct for potential variations in sample preparation and instrument performance. For the study samples (case and control groups), no samples were excluded based on the aforementioned QC criteria (±2 SD and CV ≤ 10%), as all detected metabolic indicators of the samples met the quality control requirements. During the entire study period (June 2024–June 2025), the detection instrument, reagent model, and operating parameters remained unchanged to further guarantee the reliability and consistency of detection results across different batches.

#### 2.2.3. Statistical Analysis

Statistical analyses were performed using IBM SPSS Statistics, Version 26.0 (International Business Machines Corp., Armonk, NY, USA). Quantitative data that exhibited a normal distribution were presented as means ± standard deviation (X ± SD), whereas non-normally distributed data were represented as medians with interquartile ranges [Q50 (Q25, Q75)]. Qualitative data were summarized as frequencies and percentages (%). The Mann–Whitney U test was applied to non-normally distributed data, while normally distributed data underwent analysis via the *t*-test to identify potential factors associated with sleep disorders among two groups. For comparisons across multiple groups, normally distributed quantitative data were analyzed using one-way analysis of variance (ANOVA) followed by Bonferroni post hoc correction for pairwise comparisons, and non-normally distributed quantitative data were analyzed using the Kruskal–Wallis H test. Qualitative data among multiple groups were compared using the Chi-square test. For the univariate group comparisons of 19 metabolites, the Bonferroni method was used for multiple testing correction. Following this, a multivariate logistic regression model was developed to evaluate the relationship between the identified factors and sleep disorders, utilizing a stepwise selection approach for model construction. Receiver Operating Characteristic (ROC) curves and calibration plots were generated, in addition to Decision Curve Analysis (DCA), to assess the discriminative power and consistency of the predictive model. The optimal cut-off value of the model was determined based on the Youden’s index (J = sensitivity + specificity − 1). For threshold-specific performance analysis, a series of gradient cut-off values were selected to evaluate the diagnostic efficacy of the model across different thresholds, and the area under the curve (AUC), sensitivity, specificity and 95% confidence interval (95% CI) at each cut-off value were calculated to explore the performance variation trend of the model. The model’s internal validation was conducted through 10-fold cross-validation. A *p*-value of less than 0.05 was deemed statistically significant.

## 3. Results

### 3.1. Comparison of Metabolite Indicators Between Groups

The mean age of children in the sleep disorder group was 7.135 years (range: 4.730 to 9.940), while the control group had a mean age of 7.670 years (range: 4.670 to 10.330). There was no statistically significant difference in age, gender, and BMI between the two groups (all *p* > 0.05). Univariate analysis demonstrated significant differences in several metabolite indicators between the sleep disorder group and the control group (*p* < 0.05). Within the amino acid profile, levels of aspartic acid, tyrosine, tryptophan, glutamic acid, glutamine, glycine, serine, and phenylalanine were markedly elevated in the sleep disorder group compared to controls, whereas taurine levels were significantly reduced. Regarding fatty acid indicators, levels of ARA, DHA, LA, and cerotic acid were significantly lower in the sleep disorder group. Conversely, levels of behenic acid were significantly higher, and the ratio of cerotic acid/beheric acid was also significantly lower in the sleep disorder group (*p* < 0.05). No statistically significant differences were found for EPA, lignoceric acid, kynurenic acid, or GABA between the groups (Table 1).

### 3.2. Comparison of Baseline Characteristics and Serum Metabolic Indicators Among Subtypes of Sleep Disorders

This study included 143 patients with sleep disorders, classified into four groups according to the ICSD-3 criteria: insomnia (*n* = 54), parasomnia (*n* = 46), sleep-related movement disorders (*n* = 22), and sleep-disordered breathing (*n* = 21). We compared the differences in baseline characteristics and serum metabolic indicators among these subtypes. The results showed no statistically significant differences in age (F = 0.299, *p* = 0.826), gender composition (*χ*^2^ = 0.619, *p* = 0.892), or BMI (F = 0.903, *p* = 0.442) across the subtypes (all *p* > 0.05). However, there was a significant difference in taurine levels among the subtypes (F = 6.131, *p* = 0.001), with the highest levels observed in the parasomnia group (130.255 ± 44.785 μmol/L) and the lowest in the insomnia group (93.958 ± 43.784 μmol/L). No statistically significant differences were found for other metabolic indicators, such as arachidonic acid, aspartate, and glutamine, among the subtypes (all *p* > 0.05) (Table 2).

### 3.3. Logistic Regression Analysis of Factors Influencing Sleep Disorders

Indicators with *p* < 0.1 from the univariate analysis were incorporated into a multivariate logistic regression model. A stepwise selection method (stepwise) was adopted for variable screening (entry criterion: *p* < 0.05; removal criterion: *p* > 0.1). Although glutamic acid exhibited the highest diagnostic efficacy in univariate analysis (AUC = 0.831), it was excluded from the final model due to potential multicollinearity with other variables (e.g., glutamine, a direct precursor of glutamate) and lower relative contribution to the model compared to the six factors retained. The goodness-of-fit evaluation indicated a −2 log likelihood value of 161.483, with the likelihood ratio test resulting in *χ*^2^ = 201.098 (df = 6, *p* < 0.001), suggesting a good fit for the model. McFadden’s R^2^ was 0.55, and Nagelkerke’s R^2^ was 0.714, indicating strong explanatory power. Ultimately, six indicators (ARA, cerotic/behenic acid ratio, aspartic acid, glutamine, taurine, and phenylalanine) were identified as independent factors for the development of sleep disorders (*p* < 0.05) (Table 3).

### 3.4. Evaluation of Predictive Model Performance

#### 3.4.1. Primary Model Performance

**Figure 1:**
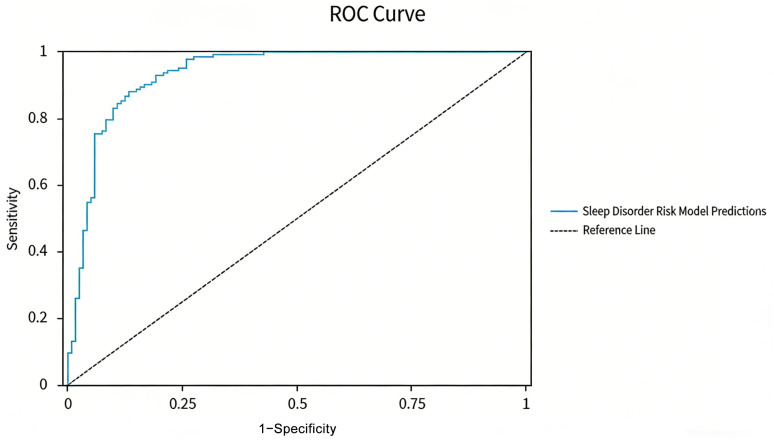
ROC curve for the sleep disorder risk prediction model. The optimal cutoff value is determined to be 0.748, with an AUC of 0.935, which indicates that the model exhibits outstanding discriminative ability. The curve’s proximity to the upper left corner further suggests that the model consistently demonstrates high sensitivity and specificity across various threshold levels.

The area under the ROC curve (AUC) for the predictive model was determined to be 0.935 (95% CI: 0.904–0.967, *p* < 0.001). At the optimal cutoff value of 0.748, the model demonstrated a sensitivity of 0.881 and a specificity of 0.867 (Table 4, Figure 1). To further assess the diagnostic superiority of the combined model, we compared the ROC curve parameters for each core predictor individually against those of the combined model (Table 5). The findings revealed that among individual indicators, glutamic acid displayed the highest diagnostic performance (AUC = 0.831, 95% CI: 0.789–0.873), followed by phenylalanine (AUC = 0.785, 95% CI: 0.738–0.832) and ARA (AUC = 0.762, 95% CI: 0.713–0.811). In contrast, taurine exhibited relatively poor diagnostic efficacy as a standalone indicator (AUC = 0.422, 95% CI: 0.361–0.483). In comparison to these individual indicators, the AUC for the model incorporating all six predictive factors significantly improved to 0.935 (95% CI: 0.904–0.967). The model’s sensitivity (0.881), specificity (0.867), positive predictive value (0.872), and negative predictive value (0.870) all exceeded those of any single indicator. This highlights the advantage of integrating multiple indicators, effectively consolidating complementary information and substantially improving diagnostic accuracy for sleep disorders in children. Moreover, this approach mitigates the risk of misclassification due to compensatory metabolic pathways or individual variances inherent in single indicators.

**Figure 2:**
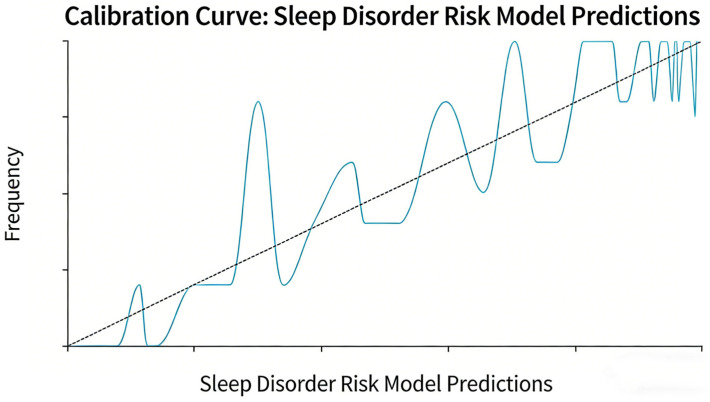
The calibration curve of the prediction model in the sample, with the solid line representing the model’s actual fitting curve and the dashed line denoting the ideal 45° line, aligns closely with the ideal scenario, exhibiting a shape that approximates a forty-five degree angle. There is a strong consistency between the model’s predicted values and the observed values. These findings suggest that the prediction model has good calibration, demonstrating a high level of agreement between its predicted probabilities of sleep disorders in patients and the actual probabilities.

**Figure 3:**
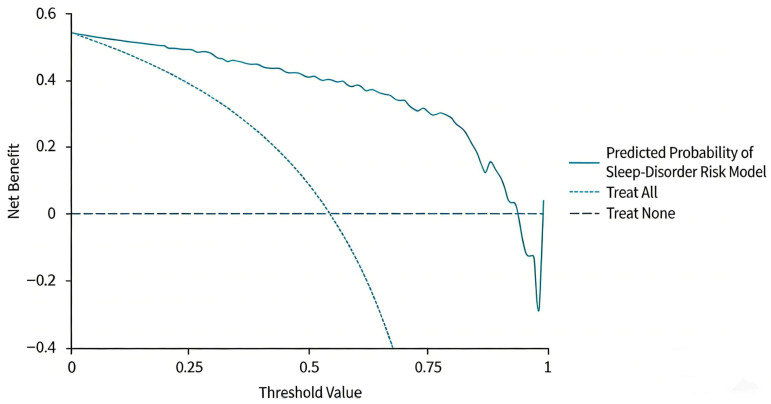
The DCA curve of the prediction model reveals that across the threshold probability spectrum of 1% to 100%, patients experience a net benefit that surpasses that of the two alternative extreme curves. This suggests that the model maintains its clinical validity within this range.

Additionally, the calibration curve closely aligned with the ideal 45° line (Figure 2), and the Hosmer–Lemeshow test produced a *p*-value exceeding 0.05, indicating good agreement between the model’s predicted probabilities and the actual incidence of sleep disorders. The DCA demonstrated that over a threshold probability range of 1% to 100%, the net benefit of the combined model consistently exceeded that of the extreme curves, which posit that all patients are either diseased or non-diseased. This indicates that the combined model could provide beneficial implications for clinical practice (Figure 3).

#### 3.4.2. Threshold Analysis

To guide clinical decision-making, we evaluated model performance across a spectrum of probability cutoffs (Table 6). At the optimal threshold of 0.748, the model achieved balanced sensitivity (88.1%) and specificity (86.7%). Lowering the threshold to 0.70 improved sensitivity to 74.6% while maintaining specificity at 86.7%, which may be preferred in screening scenarios prioritizing case detection. Further reduction to 0.60 yielded sensitivity of 71.0% and specificity of 82.4%. These findings demonstrate the model’s flexibility to accommodate different clinical priorities.

#### 3.4.3. Internal Validation

**Figure 4:**
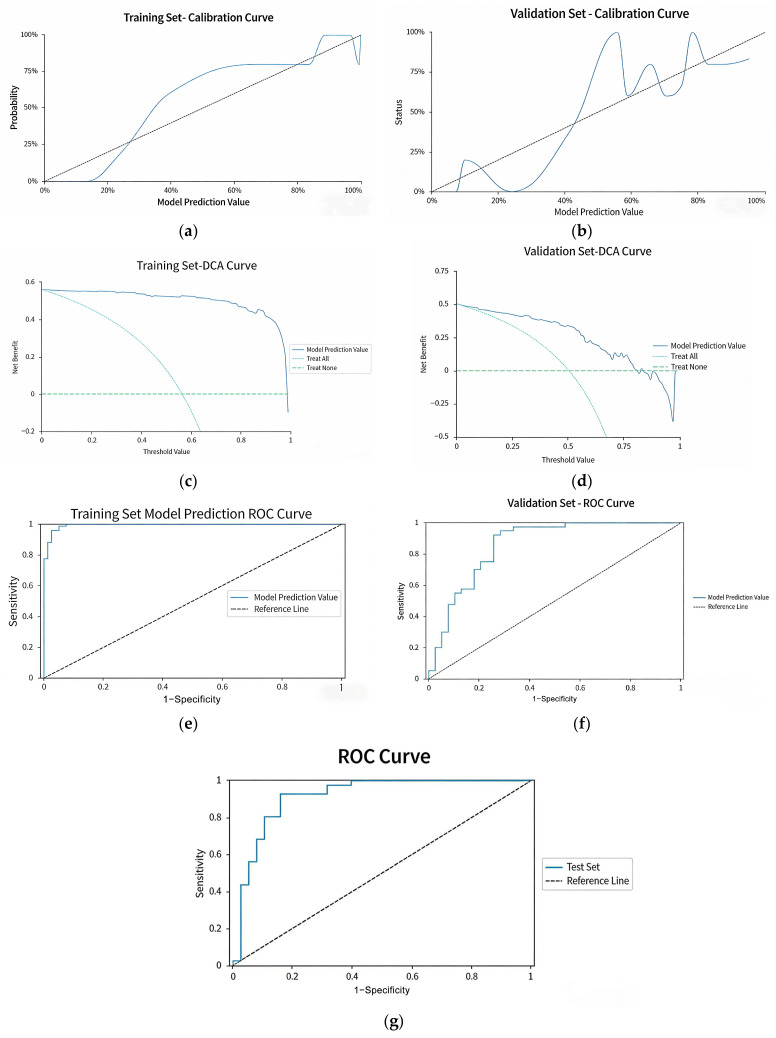
10-fold cross-validation and internal validation profiles of the risk prediction model for childhood sleep disorders (7:3 training/test set split). (**a**) Calibration curve of the training set: the solid line represents the predicted probability of the model, and the dashed line represents the ideal 45° fitting line, reflecting the consistency between the predicted and actual incidence of sleep disorders. (**b**) Calibration curve of the validation set: the interpretation is consistent with (**a**). (**c**) Decision curve analysis (DCA) of the training set: the blue solid line indicates the net benefit of the model prediction, the light blue dashed line represents the “Treat All” strategy, and the green dashed line represents the “Treat None” strategy; a net benefit higher than the two extreme curves indicates the clinical value of the model. (**d**) DCA of the validation set: the interpretation is consistent with (**c**). (**e**) Receiver operating characteristic (ROC) curve of the training set: the vertical axis is Sensitivity, the horizontal axis is 1−Specificity, and the curve close to the upper left corner indicates a higher discriminative ability of the model. (**f**) ROC curve of the validation set (derived from 10-fold cross-validation): reflecting the discriminative ability of the model in the cross-validation subset, verifying the model without overfitting; (**g**) ROC curve of the test set (derived from 7:3 independent train/test split): reflecting the diagnostic performance of the model in unknown independent samples, verifying the generalizability of the model.

In addition, our study randomly divided the total sample (*n* = 263) into a training set (*n* = 184) and a testing set (*n* = 79) in a 7:3 ratio, which were used for model construction and preliminary performance validation, respectively. A 10-fold cross-validation was further performed on the training set to evaluate the model’s generalization ability and avoid overfitting: the training set was equally split into 10 subsets, with 9 subsets used for model training and 1 subset as the validation subset in each iteration, and the process was repeated 10 times to obtain the average performance of the model. Results of 10-fold cross-validation shown in the tables and figures below (Table 7a–c, Figure 4a–g). The 10-fold cross-validation revealed that the model achieved an AUC of 0.927 in the training set and 0.916 in the independent test set (Table 7b), with an overall accuracy of 86.08%, a comprehensive precision of 86.09% and a recall of 86.08% (Table 7a), indicating excellent diagnostic performance. The Hosmer–Lemeshow test showed *p*-values >0.05 for both the training and test sets (Table 7c), and the calibration curves, DCA curves and ROC curves of the validation subset (derived from cross-validation) all exhibited good fitting effects (Figure 4a–f), confirming that the model had high calibration and no obvious overfitting. The test set ROC curve (Figure 4g) further verified the model’s stable discriminative ability in unknown independent samples.

From the table above, it can be seen that the final model achieved an accuracy of 86.08% on the test set, with a precision (overall) of 86.09%, a recall (overall) of 86.08%, and an F1-score (overall) of 0.86. The model performance is acceptable.

The AUC values for both the training set and the test set are above 0.9, indicating that the internal validation model has high diagnostic value.

The Hosmer–Lemeshow test for both the training set and the test set shows *p*-values greater than 0.05, indicating that the validation model has high calibration.

## 4. Discussion

This study successfully developed a risk prediction model for sleep disorders in children, utilizing six independent predictive factors derived from a systematic analysis of serum amino acid and fatty acid metabolic profiles. The model exhibited exceptional diagnostic performance, achieving an area under the ROC curve of 0.935. At an optimal threshold of 0.748, it demonstrated sensitivity and specificity rates of 0.881 and 0.867, respectively. This advancement offers a novel objective tool for the early detection of sleep disorders in children, effectively mitigating the limitations posed by traditional diagnostic methods. A key finding of this research is the significant metabolic disturbance of amino acids observed in children with sleep disorders. Levels of aspartate, tyrosine, tryptophan, glutamate, glutamine, glycine, serine, and phenylalanine were significantly elevated compared to the control group, whereas taurine, known for its neuroprotective properties, was notably decreased. These amino acids play critical roles as precursors in neurotransmitter synthesis and establish a complex interaction network that regulates central nervous system functions. The observed metabolic imbalance of these amino acids is viewed as a fundamental pathological basis contributing to the onset of sleep disorders in children. Prior studies have indicated that the precise regulation of the sleep–wake cycle is contingent upon the coordinated actions of various neurotransmitter systems across different brain regions. Among these systems, GABA and glutamate serve as the primary inhibitory and excitatory neurotransmitters, respectively, facilitating the transition between wakefulness and sleep [19]. From the perspective of metabolic networks and functional correlations, glutamate is the most abundant excitatory neurotransmitter in the human brain, while aspartate is metabolically closely intertwined with glutamate primarily through the malate-aspartate shuttle and transamination reactions; the two exhibit intricate metabolic synergy, and this synergy modulates glutamate concentration in an activity-dependent manner, thereby indirectly regulating the excitation threshold of the central nervous system [20,21]. In this study, the levels of aspartate and glutamate in children with sleep disorders demonstrated a synchronous increasing trend, indicating that the central nervous system may be experiencing a state of excessive excitation. This disturbance has the potential to disrupt the dynamic equilibrium of the sleep–wake cycle, corroborating previous research that identifies “excitatory–inhibitory imbalance as a fundamental pathological mechanism underlying sleep disorders.” [22].

This study confirmed via multivariate logistic regression that children with sleep disorders had significantly elevated serum levels of glutamine and glutamate, and both were identified as independent risk factors for childhood sleep disorders, consistent with the findings of Yoon S et al. who reported that excessive activation of the glutamatergic system disrupts the transition between sleep and wakefulness [23]. From a metabolic pathway standpoint, glutamine can be converted to glutamate via the catalysis of glutaminase [24], and glutamate, as the major excitatory neurotransmitter in the central nervous system, excessive glutamate release results in overactivation of glutamate receptors on the surface of neurons, triggering a sequence of detrimental responses, including amplified oxidative stress [25,26], impaired mitochondrial function [27], and disrupted calcium homeostasis [28,29]. However, it should be clarified that this study only measured the steady-state serum levels of glutamine and glutamate, without directly verifying the conversion efficiency between them or the existence of excitotoxicity. Therefore, this mechanism remains a hypothesis that requires further validation through in vitro cell experiments or metabolic flux tracing techniques in future studies.

We found that children with sleep disorders had significantly elevated levels of phenylalanine and tyrosine, the phenylalanine was identified as an independent risk factor. In the investigation of amino acid metabolism disorders, the metabolic relationship between phenylalanine and tyrosine, along with their effects on sleep, merits particular attention. Phenylalanine is converted into tyrosine through the enzymatic action of phenylalanine hydroxylase [30], and tyrosine is subsequently metabolized by tyrosine hydroxylase to yield neurotransmitters such as dopamine and norepinephrine, which play critical roles in central nervous system signaling. Previous studies involving children aged 5 to 10 years have explored the correlation between phenylalanine and sleep, indicating that abnormalities in phenylalanine metabolism specifically impact sleep anxiety, with only a limited association with overall sleep quality [31]. We hypothesize that a key mechanism underlying this association may involve the simultaneous elevation of both amino acids, potentially leading to increased synthesis of wake-promoting neurotransmitters, which could disrupt neuronal signaling, interfering with normal sleep rhythms and ultimately alter sleep architecture. Existing literature supports the notion that individuals with sleep disorders often exhibit heightened sympathetic nervous system activity [32,33]. As norepinephrine serves as a primary neurotransmitter of the sympathetic nervous system, its secretion follows a distinct circadian rhythm; nocturnal levels should be considerably lower than those during wakefulness, with concentrations during REM sleep being less than those observed during NREM sleep, progressively increasing after morning awakening. However, it should be noted that this study did not measure the levels of dopamine, norepinephrine, or other related neurotransmitters, and the proposed mechanism remains speculative. Future studies should detect central or peripheral neurotransmitter concentrations to validate this hypothesis.

Interestingly, we identified significant elevations in glycine and serine levels in children with sleep disorders. However, multifactorial regression analysis did not incorporate these two amino acids into the final predictive model, indicating their elevation may not act as direct pathogenic factors for sleep disorders. Alternatively, dietary differences could be a potential contributor. Glycine is rich in animal-derived foods such as pork, beef, poultry, fish, dairy products, and eggs, and can be found in plant-based foods including legumes and whole grains [34]. If children in the sleep disorder group had higher daily intake of these foods, it might lead to elevated serum levels. Regrettably, this study did not collect detailed dietary data, making it impossible to quantify dietary contributions or rule out this confounding factor. Additionally, altered protein metabolism may be involved. Glycine and serine levels, as key intermediates in protein synthesis and decomposition, can reflect systemic protein metabolic status [35]. As noted by Lamon S et al., acute sleep deprivation is associated with metabolic disturbances that impair protein homeostasis, specifically by reducing postprandial skeletal muscle protein synthesis [36]. However, we did not measure protein metabolism-related biomarkers to verify this hypothesis, leaving the potential link between protein metabolism and amino acid elevation unaddressed. From a metabolic perspective, glycine and serine are closely interconnected. The human enzyme serine hydroxymethyltransferase (SHMT) is composed of two isoforms, SHMT1 and SHMT2. SHMT1 primarily catalyzes the conversion of serine to glycine, while SHMT2 serves dual functions in both the interconversion of these amino acids and the cleavage of serine [37,38]. Physiologically, muscle relaxation during REM sleep relies on the synergistic inhibitory effects of GABA and glycine on motor neurons [39]. Elevated glycine and serine may reflect a compensatory mechanism, though dietary differences or altered protein metabolism are equally plausible. However, this hypothesis lacks direct evidence: this study did not measure the inhibitory effects of glycine/serine on excitatory neurons or verify the regulatory relationship between glycine/serine levels and excitatory amino acid concentrations. Additionally, the failure of these two amino acids to emerge as independent risk factors suggests that none of these mechanisms-compensatory, dietary, or metabolic-can be confirmed from our data. Future studies should address these gaps by collecting detailed dietary data, detecting protein metabolism markers, and validating the compensatory hypothesis through in vitro electrophysiological experiments or in vivo neurotransmitter activity assays.

On the other hand, our study found that children with sleep disorders had significantly elevated serum tryptophan levels, while kynurenine, a key intermediate in the kynurenine pathway, did not show a statistically significant difference. Traditional views hold that tryptophan is a precursor for melatonin synthesis, and its increased levels should promote sleep [40,41], but the results of this study contradict this notion. Elevated tryptophan with unchanged kynurenine does not support enhanced kynurenine pathway flux. We offer alternative speculative explanations: reduced melatonin synthesis, peripheral-tissue compartmentalization, or altered tryptophan utilization via unmeasured pathways. These hypotheses remain untested as melatonin, quinolinic acid, and metabolic flux were not assessed. It should be emphasized that this study did not measure tryptophan metabolic flux, downstream metabolites of the kynurenine pathway, or melatonin levels, and the lack of significant differences in kynurenine levels means this hypothesis lacks direct evidence. Future studies should employ 13C-labeled metabolic tracing to clarify tryptophan’s metabolic fate in children with sleep disorders, while concurrently measuring serum melatonin and key kynurenine pathway downstream metabolites such as quinolinic acid to verify this hypothesis.

In addition to their central roles as neurotransmitter precursors, the detected serum amino acids exert systemic functions linked to chronic stress-a key feature of chronic sleep disorders [42,43]. For example, tryptophan is involved in the synthesis of melatonin and serotonin via the indoleamine pathway, and its metabolite levels increase in response to sleep deprivation [44]; meanwhile, taurine levels are significantly elevated in blood and urine during sleep restriction, as consistently observed in metabolomic analyses of sleep disturbance, while taurine ameliorates chronic stress-associated neurophysiological abnormalities and modulates oxidative stress responses [45]. In addition to their central role as neurotransmitter precursors, the serum amino acids examined in this study (such as tryptophan and taurine) also possess functions related to chronic stress. Their metabolic changes may influence sleep. Subsequent research should focus on validating these findings through metabolic flux tracing and examining correlations between central and peripheral metabolic markers.

Another noteworthy aspect is the metabolic dysregulation of taurine. This vital inhibitory neurotransmitter signaling molecule, present in tissues and blood, plays an essential role not only in regulating the metabolism of sleep-related factors such as glycine and GABA [46,47] but also exhibits multifaceted neuroprotective effects. These include promoting brain cell proliferation, providing antioxidant defense against damage, and protecting against toxic insults [48]. Our study revealed that children with sleep disorders had markedly reduced levels of taurine. We further analyzed the subtypes of sleep disorders and showed significant differences in taurine levels among them, with the highest level in the parasomnia group and the lowest in the insomnia group. Although the AUC for taurine as a single diagnostic marker was merely 0.422, indicating limited diagnostic value, its deficiency as a key neuroprotective factor carries substantial pathological implications. Prior research has established that taurine can augment the central depressant effects of substances like ethanol and pentobarbital, thereby extending sleep duration. The observed decline in taurine levels may directly impair the body’s capacity to inhibit excitability within the central nervous system, significantly elevating the risk of developing sleep disorders [49]. It’s a pity that we did not measure the inhibitory effect of taurine on central nervous system excitability or verify the association between taurine deficiency and excitatory–inhibitory imbalance. Therefore, this hypothesis requires further validation through electrophysiological studies or neurotransmitter balance detection in future research.

In this study, alongside exploring amino acid metabolism, we found that serum ARA levels were significantly lower in children with sleep disorders, and multivariate logistic regression analysis identified ARA as an independent protective factor for childhood sleep disorders. Existing studies indicating that ARA is essential for maintaining neuronal membrane fluidity and synaptic integrity [50]. Diminished levels of ARA can adversely impact cellular membrane physiology and the efficiency of synaptic signaling [51]. More critically, ARA directly influences pivotal molecules involved in sleep regulation within the central nervous system—specifically, the large conductance calcium-activated potassium (BK) channels. ARA enhances the activity of these channels by up to fourfold through direct interactions with channel proteins. BK channels are indispensable for the regulation of sleep–wake cycles and play essential roles in cardiac neural control [51]. These findings provide a theoretical basis for the hypothesis that ARA deficiency may contribute to the pathogenesis of sleep disorders by impairing BK channel activity, disrupting neuronal membrane integrity, or destabilizing inflammatory homeostasis. However, it should be clarified that we only measured serum ARA levels and did not directly verify its effects on BK channel activity, neuronal membrane function, or inflammatory markers. Thus, this mechanism remains speculative and requires further validation through in vitro channel activity assays or in vivo animal models in future studies. Furthermore, we also found that DHA, LA, and cerotic acid were significantly lower than those observed in the control group, while behenic acid levels were considerably higher. Additionally, there was a marked reduction in the ratio of cerotic acid to behenic acid. Interestingly, beyond neuronal membrane function, serum lipids and branched-chain amino acids are closely involved in sleep-related inflammation and energy metabolism. Sleep disorders are consistently linked to perturbed lipid metabolism and altered amino acid catabolism, associated with activated inflammation and mitochondrial fatty acid oxidation dysfunction [42,44]. Pawan K. Jha et al. have pointed out that polyunsaturated fatty acids, as well as amino acids like taurine, threonine, and tryptophan, can respond to oxidative stress or stress hormones. This response is closely related to the pathological processes of sleep disorders. They participate in the regulatory feedback of oxidative stress and stress hormones by modulating neurotransmitter metabolism, antioxidant balance, and lipid homeostasis [52].

Moreover, both cerotic acid and behenic acid are long-chain saturated fatty acids involved in lipid metabolism and cell membrane formation [53]. In our study, the levels of cerotic acid in the sleep disorder group were found to be decreased, while the levels of behenic acid were elevated, resulting in a significant reduction in their ratio, which was identified as an independent protective factor. From a metabolic phenotype perspective, both cerotic acid and behenic acid are very long-chain fatty acids, and their metabolic pathways are consistent, following a composite route of “ω-oxidation initiation and peroxisomal β-oxidation termination.” [54]. Within the framework of observational research, we speculate that the reduced ratio may be related to potential abnormalities in the function of the long-chain fatty acid peroxisomal β-oxidation pathway; however, this association needs further validation through β-oxidation activity testing and ratio correlation analysis. Additionally, considering that mitochondrial β-oxidation is a key pathway for brain energy supply and that the stability of the sleep–wake cycle relies on continuous energy support, this metabolic imbalance may indirectly affect sleep regulation by influencing brain energy homeostasis [55]. It is important to clarify that, due to the observational design of this study, the aforementioned mechanisms are merely reasonable hypotheses based on metabolic pathway logic and existing literature, lacking direct experimental evidence for support. Future prospective studies or in vitro experiments are needed to further validate this speculation.

The multimodal predictive model developed in this study offers notable advantages over existing diagnostic tools. First, it demonstrates strong objectivity by relying on serum metabolic indicators, mitigating subjective biases of questionnaire-based assessments and enhancing result reliability. Second, it exhibits excellent diagnostic efficacy with an AUC of 0.935, sensitivity and specificity both exceeding 85%. Rigorous internal validation (7:3 training-test split combined with 10-fold cross-validation) confirmed its stability: the 10-fold cross-validation on the training set yielded an AUC of 0.927 for the training set and 0.916 for the independent test set, with no significant performance decline, and the validation subset derived from cross-validation showed good calibration and clinical net benefit. The model achieved an overall accuracy of 86.08% with the Hosmer–Lemeshow test *p* > 0.05 for both sets, effectively reducing overfitting risk and demonstrating robust generalization ability in the internal cohort. Third, its practicality is evident as the detection indicators can be measured using standard LC-MS/MS techniques. The model’s calculations are straightforward and do not necessitate specialized equipment, making it well-suited for implementation in primary healthcare settings and large-scale screening initiatives. Furthermore, this research incorporates both amino acid and fatty acid metabolic indicators, highlighting the multifaceted metabolic disturbances associated with sleep disorders, thus offering a more comprehensive perspective for mechanistic investigations. Beyond these strengths, the model’s adjustable threshold provides additional clinical flexibility. The predictive model demonstrates robust performance across various threshold settings, allowing clinicians to adjust the cutoff based on specific clinical scenarios. For instance, a lower threshold may be adopted in primary screening settings to maximize case detection, while a higher threshold could be used for confirmatory diagnosis. This adaptability enhances the model’s practical utility in diverse healthcare contexts.

However, this study has several limitations. First, the primary limitation of this study is the absence of model validation using independent external cohorts. While internal validation was performed through 10-fold cross-validation, which confirmed the model’s good internal stability, it does not evaluate its generalizability across heterogeneous populations, nor does it facilitate the translation of the model into clinical practice. Future research should focus on conducting multicenter external validation with independent cohorts from 3 to 5 tertiary hospitals, emphasizing the verification of the model’s discriminative ability, calibration, and clinical net benefit. This will help clarify its applicability and potential for broader adoption. The case–control design may introduce selection bias. All participants were recruited from a single pediatric health care clinic, which limits the generalizability of the results to other populations or regions. For instance, regional differences in children’s nutritional status, lifestyle, or medical-seeking behavior could affect metabolic profiles and sleep disorder prevalence. Second, we lacked quantitative severity grading for sleep disorders. No CSHQ were adopted to conduct stratified evaluation of the clinical severity of sleep disorders in the case group, and the retrospective study design also did not include the setting of severity grading indicators for sleep disorders. As a result, we cannot characterize the relationship between metabolic biomarker levels and sleep disorder severity or assess the predictive model’s performance across mild, moderate, and severe cases. Future prospective studies should use standardized severity metrics to facilitate dose–response analyses. In addition, no age, gender or BMI matching was conducted between the two groups given the natural outpatient recruitment, while statistical analyses confirmed no significant baseline differences in these indicators between the two cohorts, potential residual confounding from such factors, however, cannot be fully ruled out. Third, as a retrospective study based on routine clinical data, we did not collect detailed information on potential confounders such as dietary patterns, protein metabolism markers, family sleep environment, and daily physical activity, which may indirectly influence serum amino acid levels and hinder the clarification of whether glycine/serine elevation is a compensatory response, dietary consequence, or result of altered protein metabolism. Future prospective studies will incorporate standardized dietary assessments. Fourth, while the sample size was sufficient for preliminary model construction, future studies could expand this to enhance the robustness of the predictive model.

## 5. Conclusions

This study successfully established a risk prediction model for childhood sleep disorders based on six serum metabolic indicators—ARA, the ratio of cerotic acid to behenic acid, aspartic acid, glutamine, taurine, and phenylalanine. The model demonstrates excellent discriminative ability, robust generalizability via cross-validation, and flexible threshold adaptability for diverse clinical scenarios. Subgroup analysis of four sleep disorder subtypes showed that only taurine levels differed significantly, while other metabolic indicators were consistent across subtypes. The model’s objective results and ease of use render it an effective tool for early screening of pediatric sleep disorders, providing valuable guidance for precise clinical interventions with significant potential for broader clinical application. Additionally, this study enhances the understanding of the pathological mechanisms underlying sleep disorders in children from a metabolomics perspective, proposing a theoretical framework linking metabolic dysregulation, neuronal excitation, inhibition imbalance, and sleep disorders, thereby establishing a foundation for targeted metabolic interventions as innovative therapeutic approaches.

## Figures and Tables

**Table 1:** Comparison of amino acid and fatty acid indicators between the two groups.

	Control Group (*n* = 120)	Sleep Disorders Group (*n* = 143)	*t*/*z*/*χ*^2^-Value	*p* *	Cohen’s d
Age, years	7.135 (4.730, 9.940)	7.670 (4.670, 10.330)	0.199	0.842	0.012
Gender, *n* (%)			2.412	0.12	-
Male	53 (44.167%)	78 (54.545%)			
Female	67 (55.833%)	65 (45.455%)			
BMI (kg/m^2^)	16.615 ± 1.732	16.746 ± 1.657	0.626	0.532	0.078
Glutamine	573.502 ± 80.711	649.307 ± 92.729	7.002	0.000	0.867
Tryptophan	58.204 ± 11.479	64.254 ± 12.222	4.11	0.000	0.509
LA	98.435 (60.690, 140.415)	61.610 (42.860, 82.330)	−6.17	0.000	0.804
EPA	0.555 (0.380, 0.778)	0.450 (0.300, 0.750)	−1.913	0.056	0.191
DHA	18.340 (13.933, 25.293)	13.050 (9.290, 17.370)	−5.447	0.000	0.606
ARA	10.240 (7.582, 15.260)	6.710 (5.600, 8.000)	−8.648	0.000	1.316
Lignoceric acid	0.220 (0.103, 0.760)	0.280 (0.200, 0.370)	1.816	0.069	0.19
Behenic acid	0.240 (0.113, 1.433)	0.330 (0.240, 0.430)	2.287	0.022	0.46
Cerotic acid	0.275 (0.100, 0.800)	0.200 (0.120, 0.260)	−3.277	0.001	0.743
Cerotic/behenic acid	0.635 (0.480, 2.018)	0.540 (0.400, 0.710)	−3.477	0.001	0.463
Aspartic acid	23.525 (20.090, 28.725)	33.220 (28.660, 40.330)	9.065	0.000	1.048
Tyrosine	67.875 (58.712, 77.310)	81.670 (65.480, 96.880)	4.832	0.000	0.581
Glutamic acid	53.150 (44.985, 73.895)	88.030 (74.830, 108.560)	9.242	0.000	0.946
Glycine	250.590 (219.697, 277.525)	304.190 (248.010, 359.840)	5.573	0.000	0.468
Serine	154.600 (146.155, 172.550)	169.050 (152.560, 195.200)	4.092	0.000	0.481
Kynurenine	1.230 (1.083, 1.390)	1.110 (0.870, 1.550)	−1.93	0.054	0.063
GABA	0.370 (0.323, 0.460)	0.420 (0.330, 0.510)	1.96	0.05	0.144
Taurine	113.545 (98.165, 133.103)	105.950 (73.890, 135.670)	−2.167	0.030	0.208
Phenylalanine	60.665 (54.645, 71.070)	88.770 (78.460, 99.130)	10.234	0.000	1.529

Note: The units for the above amino acids and fatty acids are μmol/L. For metabolite data with normal distribution (e.g., glutamine, tryptophan), the results are presented as Mean ± Standard Deviation (Mean ± SD), for non-normally distributed data (e.g., age, linoleic acid), the results are presented as Median (Interquartile Range) [Median (Q25, Q75)], where Q25 and Q75 represent the 25th and 75th percentiles, respectively. The *t*/*z* values for intergroup comparison were calculated as (Sleep Disorders Group Mean/Median) − (Control Group Mean/Median), where a positive value indicates the indicator level was higher in the sleep disorders group, and a negative value indicates the indicator level was lower in the sleep disorders group. Normally distributed data were compared by independent samples *t*-test (*t*-value), non-normally distributed data by Mann–Whitney U test (*z*-value), and categorical data (gender) by *χ*^2^ test (*χ*^2^-value). *p* *: in comparison of 19 metabolites, the Bonferroni method was used for multiple testing correction, resulting in a corrected significance level of α = 0.05/19 ≈ 0.0026.

**Table 2:** Baseline characteristics and serum metabolic indicators among subtypes of sleep disorders.

		Insomnia Group (*n* = 54)	Parasomnias Group (*n* = 46)	Sleep-Related Movement Disorders (*n* = 22)	Sleep-Disordered Breathing (*n* = 21)	F/*χ*^2^	*p*
Age		5.69 ± 3.39	6.69 ± 3.14	7.56 ± 3.65	7.31 ± 2.58	0.299	0.826
Gender		54 (37.762)	46 (32.168)	22 (15.385)	21 (14.685)	0.619	0.892
	Male	31 (39.744)	23 (29.487)	12 (15.385)	12 (15.385)		
	Female	23 (35.385)	23 (35.385)	10 (15.385)	9 (13.846)		
BMI		16.720 ± 1.645	17.030 ± 1.621	16.582 ± 1.852	16.362 ± 1.557	0.903	0.442
LA		71.071 ± 48.007	60.340 ± 29.553	78.060 ± 34.332	69.622 ± 40.717	1.153	0.33
EPA		0.624 ± 0.368	0.447 ± 0.328	0.626 ± 0.365	0.534 ± 0.336	2.482	0.064
DHA		15.054 ± 7.187	14.051 ± 9.047	14.549 ± 8.407	15.960 ± 9.496	0.281	0.839
ARA		7.091 ± 2.054	6.692 ± 2.270	7.048 ± 1.545	7.398 ± 2.321	0.618	0.605
Lignoceric acid		0.294 ± 0.143	0.352 ± 0.297	0.326 ± 0.155	0.356 ± 0.250	0.711	0.547
Behenic acid		0.354 ± 0.135	0.385 ± 0.292	0.390 ± 0.178	0.361 ± 0.145	0.275	0.843
Cerotic acid		0.241 ± 0.155	0.191 ± 0.127	0.191 ± 0.095	0.196 ± 0.125	1.512	0.214
Cerotic/behenic acid		0.699 ± 0.299	0.566 ± 0.506	0.512 ± 0.217	0.556 ± 0.367	1.835	0.144
Aspartic acid		34.312 ± 7.745	35.554 ± 9.781	37.896 ± 19.312	36.107 ± 8.590	0.58	0.629
Tyrosine		80.369 ± 23.100	84.623 ± 20.892	78.227 ± 21.390	92.310 ± 27.898	1.783	0.153
Glutamine		654.003 ± 102.658	635.493 ± 98.980	651.479 ± 78.605	665.219 ± 61.830	0.591	0.622
Glutamic acid		90.680 ± 34.416	110.104 ± 49.330	93.705 ± 52.516	99.707 ± 23.838	1.923	0.129
Glycine		322.430 ± 89.480	339.784 ± 276.114	263.939 ± 82.673	324.321 ± 64.706	0.996	0.397
Serine		176.750 ± 29.348	180.544 ± 37.897	165.783 ± 26.664	169.606 ± 26.612	1.347	0.262
Kynurenine		1.182 ± 0.472	1.319 ± 0.432	1.325 ± 0.470	1.081 ± 0.454	1.841	0.143
GABA		0.414 ± 0.137	0.436 ± 0.135	0.415 ± 0.157	0.410 ± 0.121	0.304	0.822
Taurine		93.958 ± 43.784	130.255 ± 44.785	101.132 ± 38.679	113.103 ± 44.013	6.131	0.001
Tryptophan		64.625 ± 12.928	64.405 ± 11.149	60.795 ± 9.273	66.591 ± 15.113	0.86	0.464
Phenylalanine		86.641 ± 13.383	93.020 ± 13.381	86.817 ± 23.267	91.995 ± 17.265	1.727	0.164

Note: The units for the above amino acids and fatty acids are μmol/L.

**Table 3:** Results of multivariate logistic regression analysis for sleep disorder.

Item	Regression Coefficient	Standard Error	*z* Value	Wald *χ*^2^	*p* Value	OR Value	OR Value, 95% CI
ARA	−0.288	0.074	−3.917	15.341	0.000	0.75	0.649~0.866
Cerotic/behenic acid	−0.943	0.377	−2.499	6.244	0.012	0.39	0.186~0.816
Aspartic acid	0.096	0.029	3.323	11.042	0.001	1.1	1.040~1.164
Glutamine	0.009	0.002	3.889	15.125	0.000	1.009	1.004~1.014
Taurine	−0.016	0.006	−2.814	7.918	0.005	0.985	0.974~0.995
Phenylalanine	0.046	0.015	3.163	10.008	0.002	1.047	1.018~1.078

Note: Dependent variable = status; McFadden R^2^ = 0.55; Cox & Snell R^2^ = 0.534; Nagelkerke R^2^ = 0.714. The units for the above amino acids and fatty acids are μmol/L.

**Table 4:** Summary of ROC results and AUC values.

Title	AUC	Standard Error	*p*	95% CI	Optimal Cut-Off	Sensitivity	Specificity	Cut-Off
Predicted values of the sleep disorder risk model	0.935	0.016	0.000	0.904~0.967	0.748	0.881	0.867	0.561

**Table 5:** Efficacy of each indicator for early identification of sleep disorder.

Item	AUC	Standard Error	*p*	95% CI	Optimal Cut-Off	Sensitivity	Specificity	Cut-Off
Combined diagnosis	0.939	0.016	0.000	0.907~0.971	0.764	0.972	0.792	0.349
LA	0.279	0.032	0.000	0.217~0.341	0.007	0.007	1	264.21
DHA	0.305	0.032	0.000	0.242~0.368	0.004	0.021	0.983	40.23
ARA	0.19	0.027	0.000	0.138~0.242	0	0	1	20.88
Behenic acid	0.582	0.04	0.022	0.503~0.661	0.332	0.965	0.367	0.15
Cerotic acid	0.383	0.038	0.001	0.308~0.458	0.098	0.923	0.175	0.06
Cerotic acid/Behenic acid	0.376	0.036	0.001	0.306~0.445	0.037	0.937	0.1	0.25
Aspartic acid	0.825	0.026	0.000	0.773~0.876	0.542	0.825	0.717	27.32
Tyrosine	0.673	0.033	0.000	0.608~0.738	0.332	0.615	0.717	74.6
Glutamine	0.732	0.031	0.000	0.672~0.792	0.356	0.839	0.517	563.13
Glutamic acid	0.831	0.027	0.000	0.779~0.883	0.58	0.93	0.65	60.94
Glycine	0.7	0.033	0.000	0.636~0.764	0.404	0.629	0.775	280.74
Serine	0.647	0.034	0.000	0.580~0.713	0.249	0.399	0.85	179.9
Taurine	0.422	0.035	0.030	0.353~0.492	0.101	0.168	0.933	157.22
Tryptophane	0.651	0.034	0.000	0.585~0.717	0.256	0.706	0.55	57.72
Phenylalanine	0.866	0.024	0.000	0.820~0.913	0.656	0.923	0.733	69.17

Note: The units for the above amino acids and fatty acids are μmol/L.

**Table 6:** Threshold-specific diagnostic performance analysis of the risk prediction model for childhood sleep disorders.

Threshold	AUC	Standard Error	*p*	95% CI	Optimal Cutoff Value	Sensitivity	Specificity
Predicted Values of Sleep Disorder Risk Model	0.935	0.016	<0.001	0.904~0.967	0.748	0.881	0.867
Threshold 0.748	0.825	0.021	<0.001	0.784~0.866	/	0.696	0.801
Threshold 0.70	0.846	0.023	<0.001	0.802~0.891	/	0.746	0.867
Threshold 0.60	0.805	0.028	<0.001	0.751~0.860	/	0.710	0.824

**Table 7:** (**a**) Model parameter settings and evaluation metrics of the internal validation for the sleep disorder risk prediction model in children. (**b**) AUC values of the training and test sets for the sleep disorder risk prediction model in children. (**c**) Hosmer–Lemeshow test results of the training and test sets for the sleep disorder risk prediction model.

**(a)**			
**Name**	**Parameter Name**		**Parameter Value**
Model parameter Settings	Training set ratio		0.7
	K-fold cross-validation		10% off
	Optimization algorithm		Lbfgs
	Regularization		L2
	Set intercept		yes
	Maximum number of iterations		100
	Model convergence parameters		0.001
Model evaluation effect	Accuracy rate		86.08%
	Precision (Comprehensive)		86.09%
	Recall rate (Comprehensive)		86.08%
	f1-score		0.861
**(b)**			
**AUC Metric Value**			
Training set			0.927
Test set			0.916
**(c)**			
**Group**	***χ* ^2^**	**Degree of Freedom (df)**	***p*** **Value**
Training set	2.222	8	0.973
Test set	7.342	8	0.5

## Data Availability

The data that support the study findings are available from the corresponding author on reasonable request.

## Supplementary Materials

The following supporting information can be downloaded at: https://www.mdpi.com/article/10.3390/biomedicines14030546/s1, File: Supplementary Materials.
