# Supplementary material for: Serum Amino Acid and Fatty Acid Metabolites as Predictors of Sleep Disorders in Children: A Risk Prediction Model
Source: Biomedicines. 2026 Feb 27;14(3):546. doi: 10.3390/biomedicines14030546 (PMC13023480; doi:10.3390/biomedicines14030546)
Supplement: Supplementary file 1 [file biomedicines-14-00546-s001.zip › biomedicines-4092092-supplementary.pdf]

**Supplementary Materials**

*Amino Acid Detection*

Fifty microliters of serum were placed in a 1.5 mL centrifuge tube, followed by the addition of 200 μL of methanol solution containing internal standards (20 mg/mL dithiothreitol and 0.1% formic acid). The mixture was thoroughly vortexed and subsequently centrifuged at 4° C at 14,000 rpm for 10 minutes. Two hundred microliters of the supernatant were transferred to a 2 mL centrifuge tube and dried under nitrogen at 40° C. The dried samples were reconstituted in 100 μL of mobile phase (a mixture of solvent A and solvent B in a 1:2.5 ratio, containing 70% acetonitrile), vortexed for 3 minutes, and again centrifuged at 4° C at 14,000 rpm for 10 minutes. The resulting supernatant was filtered through a 0.2 μm filter to obtain the sample for analysis, which was then transferred to an injection vial for subsequent targeted metabolomics analysis. Chromatographic separation was performed using an ACQUITY UPLC BEH amide column (1.7 μm, 2.1 mm × 100 mm, Waters Corporation, Elstree, UK). Mobile phase A consisted of 0.1% formic acid, 10 mmol ammonium formate, and a mixture of acetonitrile/water (20:80); mobile phase B comprised 0.1% formic acid, 10 mmol ammonium formate, and acetonitrile/water (10:90). The column temperature was maintained at 45° C, with an injection volume of 3 μL and a flow rate of 400 μL/min. Positive ion mode electrospray ionization was utilized, employing scheduled multiple reaction monitoring (MRM) to acquire high-quality full-scan sub-ion spectra. The mass spectrometry conditions included an ion source temperature of 550° C and an electrospray voltage of 5500 V. Optimal mass spectrometry acquisition conditions and corresponding MRM ion pairs for each amino acid were determined using single standard solutions, allowing for qualitative identification based on the retention times and characteristic ion pairs of the analytes and their internal standards.

*Fatty Acid Detection*

Fifty microliters of serum were placed in a 1 mL 96-well plate, and 200 μL of a precipitating agent containing internal standards (methanol: n-hexane = 4:1, with 0.05% formic acid) was added. The mixture was thoroughly vortexed, followed by the addition of 400 μL of n-hexane, and mixed again. The samples were then centrifuged at 10° C at 3,000 rpm for 10 minutes. Using a multichannel pipette (with tip holder), the supernatant was transferred to a 400 μL 96-well plate, and the solvent was gently evaporated under nitrogen.

The residue was reconstituted in 100 μL of 85% methanol-water solution, mixed thoroughly, and 3 μL of the resulting solution was used for analysis. Chromatographic separation was performed using an ACQUITY UPLC BEH C18 column (1.7 μm, 2.1 mm × 50 mm, Waters Corporation, Elstree, UK). Mobile phase A consisted of 0.1% ammonium hydroxide, 10 mmol ammonium formate, and acetonitrile/water (10:90); mobile phase B comprised 0.1% ammonium hydroxide, 10% isopropanol, and 90% acetonitrile. The column temperature was maintained at 35° C, with an injection volume of 3 μL and a flow rate of 400 μL/min. Negative ion mode electrospray ionization was utilized, employing scheduled MRM to acquire high-quality full-scan sub-ion spectra. Mass spectrometry conditions included an ion source temperature of 400° C and an electrospray voltage of -4500 V.

Schematic Framework of Serum Metabolite Marker Screening and Risk Prediction Model Construction for Childhood Sleep Disorders

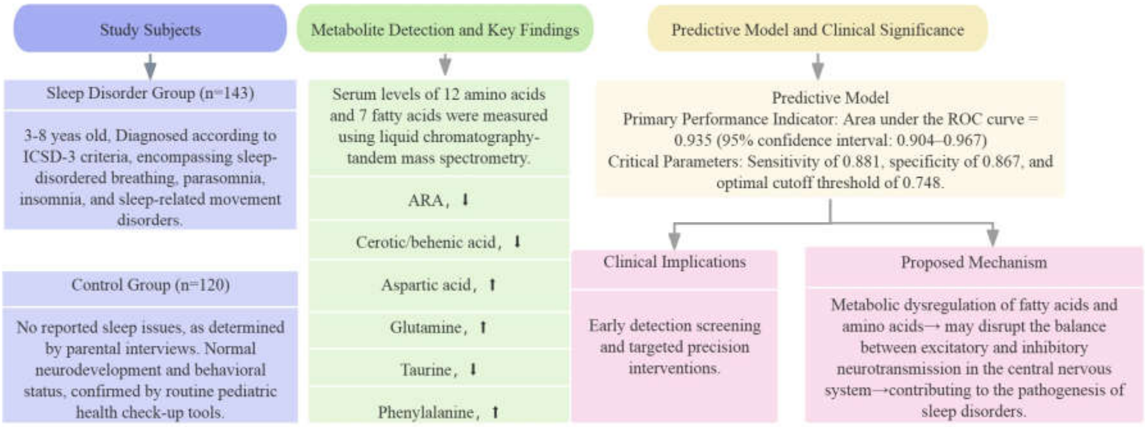
Study Subjects
Sleep Disorder Group (n=143)
3-8 yeas old, Diagnosed according to ICSD-3 criteria, encompassing sleep-disordered breathing, parasomnia, insomnia, and sleep-related movement disorders.
Control Group (n=120)
No reported sleep issues, as determined by parental interviews. Normal neurodevelopment and behavioral status, confirmed by routine pediatric health check-up tools.
Metabolite Detection and Key Findings
Serum levels of 12 amino acids and 7 fatty acids were measured using liquid chromatography-tandem mass spectrometry.
ARA，↓
Cerotic/behenic acid，↓
Aspartic acid，↑
Glutamine，↑
Taurine，↓
Phenylalanine，↑
Predictive Model and Clinical Significance
Predictive Model
Primary Performance Indicator: Area under the ROC curve = 0.935 (95% confidence interval: 0.904–0.967)
Critical Parameters: Sensitivity of 0.881, specificity of 0.867, and optimal cutoff threshold of 0.748.
Clinical Implications
Early detection screening and targeted precision interventions.
Proposed Mechanism
Metabolic dysregulation of fatty acids and amino acids→ may disrupt the balance between excitatory and inhibitory neurotransmission in the central nervous system→contributing to the pathogenesis of sleep disorders.
